# Comorbidities as a driver of the excess costs of community-acquired pneumonia in U.S. commercially-insured working age adults

**DOI:** 10.1186/1472-6963-12-379

**Published:** 2012-10-31

**Authors:** Daniel Polsky, Machaon Bonafede, Jose A Suaya

**Affiliations:** 1 Perelman School of Medicine and the Wharton School, University of Pennsylvania, Philadelphia, PA, USA; 2 Outcomes Research, Thomson Reuters, Andover, MA, USA; 3 GlaxoSmithKline Vaccines, Philadelphia, PA, USA; 4 University of Pennsylvania, Division of General Internal Medicine, Philadelphia, PA, USA

**Keywords:** Community-acquired pneumonia (CAP), Medical cost, Productivity cost, Economic evaluation, Asthma, Diabetes, Chronic Obstructive Pulmonary Disease (COPD), Congestive Heart Failure (CHF)

## Abstract

**Background:**

Adults with certain comorbid conditions have a higher risk of pneumonia than the overall population. If treatment of pneumonia is more costly in certain predictable situations, this would affect the value proposition of populations for pneumonia prevention. We estimate the economic impact of community-acquired pneumonia (CAP) for adults with asthma, diabetes, chronic obstructive pulmonary disease (COPD) and congestive heart failure (CHF) in a large U.S. commercially-insured working age population.

**Methods:**

Data sources consisted of 2003 through 2007 Thomson Reuters MarketScan Commercial Claims and Encounters and Thomson Reuters Health Productivity and Management (HPM) databases. Pneumonia episodes and selected comorbidities were identified by ICD-9-CM diagnosis codes. By propensity score matching, controls were identified for pneumonia patients. Excess direct medical costs and excess productivity cost were estimated by generalized linear models (GLM).

**Results:**

We identified 402,831 patients with CAP between 2003 through 2007, with 25,560, 32,677, 16,343, and 5,062 episodes occurring in patients with asthma, diabetes, COPD and CHF, respectively. Mean excess costs (and standard error, SE) of CAP were $14,429 (SE=44) overall. Mean excess costs by comorbidity subgroup were lowest for asthma ($13,307 (SE=123)), followed by diabetes ($21,395 (SE=171)) and COPD ($23,493 (SE=197)); mean excess costs were highest for patients with CHF ($34,436 (SE=549)). On average, indirect costs comprised 21% of total excess costs, ranging from 8% for CHF patients to 27% for COPD patients.

**Conclusions:**

Compared to patients without asthma, diabetes, COPD, or CHF, the excess cost of CAP is nearly twice as high for patients with diabetes and COPD and nearly three times as high for patients with CHF. Indirect costs made up a significant but varying portion of excess CAP costs. Returns on prevention of pneumonia would therefore be higher in adults with these comorbidities.

## Background

Community-acquired pneumonia (CAP) ranks among the top causes of death and hospitalization and it is a major driver of health care utilization and cost. Annually, there are 2 to 3 million CAP cases in the US resulting in half a million hospitalizations, 10 million physician visits, and 45,000 deaths [1]. A recent study of US non-elderly adults estimated that CAP was associated with annual excess medical costs of $8.5 billion and a productivity loss of $2.1 billion [2]. While it is understood that pneumonia risks are elevated for certain groups including several common comorbidities such as asthma and COPD [3-7], the cost burden of pneumonia has not been explored within comorbidity subgroups.

We estimate the total excess cost burden of CAP as the sum of medical costs and productivity losses (work absence and short-term disability) in a large cohort of commercially insured working-age adults throughout the United States for the most common comorbidities among individuals with pneumonia: asthma, diabetes, COPD, and CHF [2,8]. In addition to being common, these comorbidities have been singled out as potential risk factors for complications and/or mortality during or following a pneumonia episode [4,9-12]. As a result, costs of pneumonia may be higher when these comorbidities are present. In this paper we explore this possibility from the perspective of employers who insure adult populations and face the burden of medical costs and costs of absenteeism and disability.

## Methods

### Data and sample selection

This project utilized two Thomson Reuters’ research proprietary databases. For estimations of medical cost, the study is based on medical and outpatient prescription drug claims of employees and dependents in the MarketScan Commercial Claims (Commercial) and Encounters database, which included 34 million covered lives in 2008. For estimations of productivity cost, the study used workplace absenteeism and short-term disability data from the Health Productivity and Management (HPM) database. The HPM database is constructed from administrative payroll system data that includes missed work, short-term disability, and worker’s compensation from some of the same employers that have provided health care claims. HPM data has been widely used in academic publications [2,13-16]. HPM data is available for 1.4 million of the employees included in the Commercial database. Both the Commercial and HPM databases contain de-identified data that are in compliance with the Health Insurance Portability and Accountability Act (HIPAA) regulations; thus this analysis was exempt from Institutional Review Board approval. We studied adults aged 18–64 years enrolled in health plans between July 31, 2002 and December 31, 2007 with at least 6-months of continuous medical and prescription coverage.

Pneumonia episodes were identified by ICD-9-CM diagnosis codes (480.xx-486.xx) in (1) a principal or non-principal diagnosis on an outpatient claim or (2) a principal diagnosis on an inpatient claim. An index date for pneumonia was defined as the date of its first diagnosis without any similar diagnosis (i.e., pneumonia) in the prior six-months.

In order to obtain comparable controls, a control group was created from a pool of controls using a nearest neighbor propensity score 1:3 case:control match without replacement. The pool of potential controls was constructed from all individuals without pneumonia. To allow pre-index group comparisons and identification of selected comorbidities, control patients had a randomly assigned index date and were required to also have 6 months of continuous medical and prescription coverage preceding the index date. The propensity score of the presence of a pneumonia diagnosis was based on a logistic regression using the following covariates: index year, quarter, age, gender, geographic region, plan capitation status, industry, length of follow-up, baseline Deyo Charlson Comorbidity Index, and baseline comorbid conditions, including asthma, diabetes, cancer, cardiovascular disease, CHF, COPD, and HIV. We used this same technique to obtain controls for the subset of patients with work absence and short-term disability data.

There was no minimum enrollment time requirement following the index dates for cases and controls. The maximum follow-up time was 12 months from the index date, depending on enrollees' data availability.

As the focus of this study was community-acquired pneumonia (CAP), we excluded from the cost analysis any health-care associated or hospital-acquired pneumonia, which was defined as principal diagnosis on an inpatient claim that had evidence of an acute-inpatient or long-term care stay in the previous 30 days.

### Cost variables

The outcome variables include three components of medical costs: inpatient, outpatient, and pharmacy; and two components of productivity costs: absenteeism costs and short-term disability costs.

Medical costs were defined as total gross payments to a provider, including deductibles, copayments, and coordination of benefits. Costs were tabulated separately for inpatient medical, outpatient medical, and outpatient pharmacy. The costs of emergency room visits that led to an inpatient visit were classified as inpatient medical; all others were classified as outpatient medical. Pharmaceutical costs equaled the actual reimbursement to the pharmacy, including the drug’s administrative dispensing fee, ingredient cost, patient portion of costs, and sales tax. A payment proxy based on procedure code and region of care by the data provider was estimated for expenditures for services provided under capitation arrangements. All medical costs were adjusted to 2008 dollars using the Medical Care-Specific Consumer Price Index.

To estimate the cost of absenteeism, the number of employer-recorded days of absence from work was multiplied by a wage constant of $41/hour. This wage is based on a previous benchmarking study of health and productivity-related costs from a sample of 32 companies across the US [17] and adjusted to 2008 US $ using the US National Wage Index [18]. To estimate costs of short-term disability, the number of short-term disability days was multiplied by a $28.70/hr wage constant as a result of the observation that average wage replacement tends to be 70% of wages while on disability. This 70% figure is consistent with previously published studies [19,20].

### Analysis

Our strategy to estimate excess costs was to use a multivariate model with cases and controls with excess costs defined as the adjusted difference in costs between cases and controls. By including a control variable for number of months of data availability following the index date, we annualize costs through a post-estimation prediction under the assumption that all subjects are followed for 12 months.

Based on a comprehensive comparison of estimation methods in earlier analyses on costs of pneumonia [2], we estimated costs using the generalized linear model (GLM) with a gamma family and a log link. Due to the distinct cost distribution for each cost outcome, separate models were estimated for total inpatient cost, ambulatory care excluding pharmacy cost, and pharmacy cost. Predicted costs were then summed in order to assess total costs. Similarly, separate models were estimated to assess absenteeism and short-term disability. For inpatient costs, absenteeism, and short-term disability, two-part models were used. The control variables for all multivariate models included demographic, clinical and cost covariates. Demographic covariates included: age, gender, insurance type, US Bureau of Census region of residence, and population density status (urban vs. rural), employee class (union vs. non-union), employment status, and employee relationship. Clinical covariates included: Deyo CCI score in pre-period, COPD, congestive heart failure, cancer, cardiovascular disease, other musculoskeletal surgery, diabetes, asthma, pre-period medication use (specifically, antibiotics, long-acting beta agonists, short-acting beta agonists, or systemic steroids), an indicator of whether there was a hospitalization in the 1 month prior to index date, and time observed for follow-up. Cost covariates (adjusted to 2008 US dollars) included: total inpatient costs in pre-period, total outpatient costs in pre-period, and total pharmacy costs in pre-period.

Separate models were estimated for all subjects and for each of the selected comorbidity subgroups (asthma, diabetes, COPD, CHF). We note these comorbidity subgroups are not mutually exclusive. A separate subgroup was defined by the absence of all four of these comorbidities. Code lists are available from the authors upon request.

## Results

We identified 402,831 patients with CAP from MarketScan Commercial Claims (Commercial) and Encounters database. The overall demographic characteristics and pre-index period clinical characteristics are shown in Table 1. The mean age of CAP patients was 47 years and 45.6% were male. The most prevalent comorbid conditions were cardiovascular disease (22.2%), diabetes (8.1%), asthma (6.3%), cancer (6.0%), and COPD (4.1%). Mean length of follow-up was 318 days. Pre-period outpatient costs were nearly $3,000. This high baseline outpatient cost is driven by the high volume of cases with comorbidities. For example, pre-period outpatient costs were over $6,000 for cases with comorbidities of COPD, diabetes, and CHF.

**Table 1:** Baseline characteristics of patients aged 18-64 years with community acquired pneumonia (CAP) and non-pneumonia controls

	**Pneumonia Cases**	**Non-Pneumonia Controls**	**Standardized Difference (%)**
Sample Size	402,831	1,203,823	Not applicable
**Sociodemographics characteristics of patients**			
Male	45.6%	45.9%	0.58
Age in years, mean (sd)	46.8 (12.2)	46.5 (12.3)	2.37
Age group (yrs.)			0.00
18-34	17.6%	17.6%	0.02
35-44	20.8%	20.9%	0.02
45-54	28.4%	28.8%	0.87
55-64	33.1%	32.7%	0.87
**Comorbid conditions in patients**			0.00
Charlson-Deyo index, mean (sd)	0.44 (1.07)	0.45 (1.02)	0.74
			
**Selected for excess cost analyses**			
Asthma	6.3%	6.0%	1.31
Diabetes	8.1%	8.6%	1.65
COPD	4.1%	2.7%	7.23
Congestive heart failure	1.3%	1.1%	1.91
**Others**			
Cardiovascular disease	22.2%	23.0%	1.93
Cancer	6.0%	6.2%	0.98
HIV/AIDS	0.3%	0.3%	0.39
Hepatitis B	0.0%	0.0%	0.12
Hepatitis C	0.4%	0.2%	2.30
Orthopedic Surgery	0.1%	0.2%	1.27
Other Musculoskeletal Surgery	2.2%	2.3%	0.98
Pregnancy	0.8%	1.0%	1.40
**Patient's follow-up in days, mean (sd)**	318.2 (97.6)	318.4 (100.1)	0.20
**Insurance plan type**			
Preferred Provider Organization (PPO)	55.0%	56.3%	2.50
Health Maintenance Organization (HMO)	20.2%	19.9%	0.80
Point of Service (POS)	10.4%	10.4%	0.16
Comprehensive	8.7%	6.8%	7.27
Other	5.6%	6.6%	4.07
**Plan Type**			
Capitated	21.7%	21.7%	0.13
**Geographic region**			
Northeast	10.6%	10.7%	0.43
North Central	27.0%	26.6%	0.90
South	41.8%	42.4%	1.13
West	20.2%	19.9%	0.68
Unknown	0.5%	0.4%	0.30
**Urban/Rural residence**			
Urban	80.6%	82.2%	4.33
Rural	19.0%	17.4%	4.29
Unknown	0.4%	0.4%	0.46
**Industry type**			
Manufacturing, Durable Goods	20.8%	20.7%	0.29
Transportation, Communications, Utilities	10.5%	10.6%	0.56
Services	6.3%	6.3%	0.23
Manufacturing, Nondurable Goods	5.4%	5.5%	0.54
Finance, Insurance, Real Estate	5.3%	4.9%	1.88
Retail Trade	4.5%	4.9%	2.05
Oil & Gas Extraction, Mining	0.8%	0.8%	0.36
Unknown	46.4%	46.2%	0.45
**Dependent status**			
Employee	63.3%	62.3%	2.09
Dependent	36.7%	37.7%	2.09
**Pre-period Expenditures**			
Total inpatient costs	$1,005 ($10,752)	$1,078 ($9,746)	0.71
Total outpatient costs	$2,927 ($12,033)	$1,896 ($7,291)	10.36
Total outpatient pharmacy costs	$1,055 ($2,318)	$686 ($1,704)	18.14

Abbreviations: *sd*, standard deviation.

Demographic characteristics were similar between CAP patients and their 1,425,045 matched controls as the standardized differences were typically in the 1-3% range. The highest standardized differences were COPD at 7.29, comprehensive insurance at 7.39, and pre-period outpatient costs with standardized differences above 10; multivariate models were used to control for these remaining imbalances.

Annualized adjusted excess total medical costs of a case of CAP were estimated at $11,395 (SE=$42) across all patients, as determined by the difference between estimated costs for patients with CAP ($17,640 (SD=$26,706)) and the control patients ($6,245 (SD=$16,189)). These estimates come from costs defined in 2008 dollars as total gross payments to a provider, including deductibles, copayments, and coordination of benefits.

Excess medical costs of CAP among patients with COPD, diabetes, and CHF are higher than the average excess medical costs of CAP. Pneumonia is most costly among CHF patients at $31,593 (SE=$531). Excess costs are also substantially higher for patients with COPD ($17,039 (SE=$186)) and diabetes ($16,351 (SE=$165)). Among the comorbidities of interest, CAP with asthma had the lowest total excess medical costs at $10,158 (SE=$109). All of these costs were higher than the excess costs of CAP for patients without CHF, COPD, diabetes, or asthma ($9,866 (SE=$43)).

The detail of medical costs by each cost component sheds light on the drivers of higher excess medical costs among the comorbidity subgroups. As shown in Table 2, excess inpatient costs were highest among CHF patients ($18,339 (SE=$163)) and lowest among asthma patients ($4,908 (SE=38). Excess outpatient costs were highest among CHF patients ($11,953 (SE=387)) and lowest among asthma patients ($4,210 (SE=62)). Excess outpatient pharmacy costs appeared more similar across the comorbidity groups, ranging from $1,040 (SE=19) for asthma patients to $1,565 (SE=29) for COPD patients.

**Table 2:** Annualized adjusted excess medical cost of community acquired pneumonia by selected comorbidity group

**Attribute**	**Pneumonia Group**		**All Patients**		**No key comorbidity**		**Comorbid group**							
							**Asthma**		**Diabetes**		**COPD**		**CHF**	
Sample size	PNA Cases		402,831		333,485		25,560		32,677		16,343		5,062	
	Controls		1,425,045		1,155,049		92,524		131,544		50,785		20,814	
Cost component														
Inpatient	PNA Cases	Mean		$5,373		$4,235		$6,884		$12,111		$12,452		$27,534
		(SD)		($6,022)		($4,689)		($6,042)		($9,363)		($8,390)		($11,604)
	Controls	Mean		$1,472		$1,093		$1,976		$3,706		$3,676		$9,195
		(SD)		($2,548)		($1,833)		($2,718)		($4,706)		($3,770)		($5,925)
	Excess cost of PNA	Mean		$3,900		$3,143		$4,908		$8,404		$8,777		$18,339
		(SE)		($9)		($8)		($38)		($52)		($66)		($163)
	As % of total medical cost			34%		32%		48%		51%		52%		58%
Outpatient	PNA Cases	Mean		$9,236		$8,089		$9,304		$14,130		$13,634		$24,713
		(SD)		($18,160)		($17,234)		($9,887)		($20,046)		($14,641)		($27,510)
	Controls	Mean		$3,441		$2,843		$5,094		$7,278		$6,937		$12,760
		(SD)		($6,765)		($6,057)		($5,413)		($10,325)		($7,449)		($14,204)
	Excess cost of PNA	Mean		$5,796		$5,246		$4,210		$6,852		$6,698		$11,953
		(SE)		($29)		($30)		($62)		($111)		($115)		($387)
	As % of total medical cost			51%		53%		41%		42%		39%		38%
Pharmacy	PNA Cases	Mean		$3,031		$2,457		$3,582		$4,800		$4,695		$5,404
		(SD)		($4,434)		($4,326)		($3,098)		($3,028)		($3,678)		($4,272)
	Controls	Mean		$1,332		$979		$2,542		$3,706		$3,130		$4,103
		(SD)		($1,949)		($1,725)		($2,198)		($2,338)		($2,452)		($3,244)
	Excess cost of PNA	Mean		$1,698		$1,477		$1,040		$1,094		$1,565		$1,301
		(SE)		($7)		($7)		($19)		($17)		($29)		($60)
	As % of total medical cost			15%		15%		10%		7%		9%		4%
Total medical	PNA Cases	Mean		$17,640		$14,781		$19,770		$31,041		$30,781		$57,652
		(SD)		($26,704)		($24,880)		($17,392)		($29,843)		($23,809)		($37,756)
	Controls	Mean		$6,245		$4,915		$9,612		$14,690		$13,742		26,059
		(SD)		($10,549)		($9,179)		($9,373)		($15,955)		($12,216)		($20,440)
	Excess cost of PNA	Mean		11,395		$9,866		$10,158		$16,351		$17,039		31,593
		(SE)		($42)		($43)		($109)		($165)		($186)		($531)

Abbreviations: *PNA*, pneumonia; *COPD*, chronic obstructive pulmonary disease; *CHF*, congestive heart failure; *SD*, standard deviation; *SE*, standard error.

Note: comorbid groups are not mutually exclusive. Cost estimates are from generalized linear regressions.

All excess cost estimates are significant at p<0.01.

As shown in Table 3, productivity costs are $3,034 (SE=$55) higher for patients with pneumonia than the controls; approximately two-thirds of this difference is driven by short-term disability costs while the remainder comes from absenteeism costs. Among the comorbidity subgroups, productivity costs were highest for COPD ($6,454 (SE=$329)) and diabetes ($5,044 (SE=$263)) and lowest for CHF ($2,842 (SE=$752).

**Table 3:** Annualized adjusted excess productivity cost of community acquired pneumonia by selected comorbidity group

**Cost component**	**Pneumonia Group**		**All Patients**		**No key comorbidity**		**Comorbid group**							
							**Asthma**		**Diabetes**		**COPD**		**CHF**	
Absenteeism	PNA	Sample size		4,489		3,940		248		220		103		34
		Mean		$8,284		$8,158		$7,601		$10,502		$13,651		$9,043
		(SD)		($2,571)		($2,362)		($3,464)		($4,627)		($7,919)		($7,962)
	Controls	Sample size		1 3,441		11,802		739		1,006		308		1 01
		Mean		$7,358		$7,233		$7,625		$8,384		$9,348		$9,181
		(SD)		($2,284)		($2,095)		($3,475)		($3,694)		($5,423)		($8,084)
	Excess cost of PNA	Mean		$926		$924		$(24)		$2,118		$4,303		$(139)
		(SE)		($38)		($38)		($220)		($312)		($780)		($1,366)
Short-term disability	PNA	Sample size		15,468		13,170		894		658		545		139
		Mean		$3,535		$2,997		$5,074		$5,124		$5,241		$7,170
		(SD)		($7,258)		($5,456)		($8,505)		($6,287)		($2,697)		($5,866)
	Controls	Sample size		4 6,325		39,470		2,664		3,004		1,626		414
		Mean		$1,427		$1,187		$1,901		$2,198		$3,089		$4,190
		(SD)		($2,929)		($2,161)		($3,186)		($2,696)		($1,590)		($3,428)
	Excess cost of PNA	Mean		$2,108		$1,810		$3,173		$2,927		$2,151		$2,981
		(SE)		($58)		($48)		($284)		($245)		($116)		($498)
Total productivity costs	PNA	Mean		$11,819		$11,155		$12,675		$15,626		$18,891		$16,213
		(SD)		($7,700)		($5,945)		($9,184)		($7,807)		($8,366)		($9,889)
	Controls	Mean		$8,785		$8,420		9,526		$10,582		$12,437		$13,371
		(SD)		($3,714)		($3,010)		($4,715)		($4,574)		($5,651)		($8,780)
	Excess cost of PNA	Mean		$3,034		$2,734		$3,149		$5,044		$6,454		$2,842
		(SE)		($55)		($45)		($272)		($263)		($329)		($752)

Abbreviations: *PNA*, pneumonia; *COPD*, chronic obstructive pulmonary disease; *CHF*, congestive heart failure; *SD*, standard deviation; *SE*, standard error.

Note: comorbid groups are not mutually exclusive. Cost estimates are from generalized linear regressions.

All excess cost estimates are significant at p<0.01 except for absenteeism in the asthma and CHF comorbid groups.

**Figure 1:**
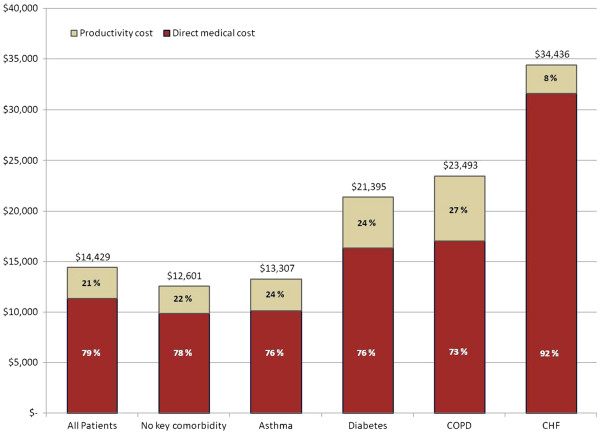
Distribution of the annualized adjusted total excess cost of pneumonia by selected comorbidity group.

Table 4 contains the adjusted overall excess cost of CAP, which are also depicted in Figure 1. CHF patients had the highest total burden of CAP, with costs nearly three times the average excess costs of CAP. CAP for patients with COPD and diabetes costs 86% and 70% more than for a typical patient. The excess cost of CAP for patients with asthma appeared similar to the average excess cost of CAP. On average, the excess cost of CAP was 79% excess direct costs and 21% excess indirect costs. The relationship between excess direct and indirect costs was similar for patients with asthma, diabetes and COPD where indirect costs accounted for 24-27% of total excess costs. Among patients with CHF, total excess costs were predominantly direct costs (92%) compared to indirect costs (8%).

**Table 4:** Annualized adjusted total excess cost of community acquired pneumonia by selected comorbidity group

**Type of excess cost**		**All Patients**	**No key comorbidity**	**Comorbid group**			
				**Asthma**	**Diabetes**	**COPD**	**CHF**
Total medical	Mean	$11,395	$9,866	$10,158	$16,351	$17,039	$31,593
	(SE)	($42)	($43)	($109)	($165)	($186)	($531)
	(95% CI)	($11,312, $11,477)	($9,782, $9,951)	($9,945, $10,371)	($16,027, $16,674)	($16,674, $17,404)	($30,553, $32,634)
Total productivity costs	Mean	$3,034	$2,734	$3,149	$5,044	$6,454	$2,842
	(SE)	($55)	($45)	($272)	($263)	($329)	($752)
	(95% CI)	($2,927, $3,141)	($2,645, $2,823)	($2,616, $3,681)	($4,528, $5,561)	($5,810, $7,098)	($1,369, $4,316)
Total cost	Mean	$14,429	$12,601	$13,307	$21,395	$23,493	$34,436
	(SE)	($44)	($44)	($123)	($171)	($197)	($549)
	(95% CI)	($14,343, $14,514)	($12,514, $12,687)	($13,066, $13,548)	($21,061, $21,730)	($23,106, $23,880)	($33,360, $35,511)
Total cost relative to patients without selected comorbidities		1.15	1.00	1.06	1.70	1.86	2.73

Abbreviation: *COPD*, chronic obstructive pulmonary disease; *CHF*, congestive heart failure; *SE*, standard error; *CI*: confidence interval.

Note: comorbid groups are not mutually exclusive. Cost estimates are from generalized linear regressions.

All excess cost estimates are significant at p<0.01.

## Discussion

The objective of this study was to estimate, for selected comorbidities, the excess health care costs and employer productivity costs of working age adults with CAP. The excess costs per case of CAP in the working-age adult population was $14,429 overall, but could be nearly 3 times as high among patients with CHF and around 75% higher among patients with COPD and diabetes. When considering the reduced cost burden from prevention of CAP, it is not sufficient to consider the risks of acquiring pneumonia, the fact that the cost per case can vary among different risk groups must be taken into account. As a result, we have provided key estimates of the burden of disease among key comorbidity risk groups.

The cost per CAP patient is highest for patients with CHF ($34,436) and is substantially higher than the cost of CAP for patients with other conditions such as COPD ($23,439), diabetes ($21,395) and asthma ($13,307), and particularly those without any of these selected comorbidities ($12,601). The higher costs, particularly for CHF and even for COPD may be explained by the clinical evidence which suggests that the pre-existing condition has a detrimental effect on pneumonia prognosis [10,21], but there is also evidence that pneumonia contributes to an exacerbation of the original condition [9,22]. These estimates can be used to better understand the cost of preventing a case of CAP, which would clearly be higher among patients with CHF than if prevention was driven only by the risks of being diagnosed with pneumonia.

With no other comorbidity-specific estimates of pneumonia costs, these results cannot be directly compared to other studies in the literature, but the overall estimates are in line with the literature. For example, our work suggests that productivity costs are about 20% of total burden. The one previous analysis that included productivity costs suggested that they represent 29% of total cost [22]. Colice et al. performed a retrospective claims-based analysis of CAP based on employer-payments for medical care made by a single Fortune 100 company in the time period from 1996 to 1998 [23]. This analysis found that the mean cost for an inpatient episode of pneumonia was $8,942, while an ambulatory CAP episode cost $466. The discrepancy in CAP costs between the two studies is likely due, in large part, to the escalation of health care expenditures over the previous decade and the cost time horizon.

A recent European study of patients with CAP reported no difference in median hospitalization costs between patients with and without diabetes, CHF, or COPD [24]. The analysis did not include outpatient costs or a non-CAP comparison cohort. The study reported a significant lack of literature describing the impact of comorbidities on CAP-related costs. Bartolome [25] and Niederman [26] evaluated age as a risk factor for higher CAP-related costs. Reyes [24], Bauer [27], and Kaplan [28] all reported higher pneumonia-related costs associated with either more severe CAP cases or in the presence of pneumonia-related complications, such as pleural effusion or respiratory failure.

The analyses described herein have limitations. While the MarketScan Research Databases represent a wide variety of nationally-representative employed patients and their dependents, it is not a random sample. As a result, the study results may not be generalizable to groups beyond the commercially insured, such as Medicaid beneficiaries or unemployed patients. Misclassification of pneumonia due to inaccurate or absent diagnosis coding is possible when analyzing claims data and basing the pneumonia diagnosis on ICD-9 codes alone and not on review of medical records or X-ray results. For example, it is possible that some mild cases were coded as “respiratory infection” and a few severe cases were coded as “sepsis”. Likewise, the intention of this analysis was to describe the burden of CAP separate from HAP; our ability to differentiate CAP from HAP may be limited by information available in an administrative claims database. Including HAP cases in our cohort of CAP patients could lead to an overestimation of the burden of CAP. However, to reduce the possibility of including HAP, we excluded from the analysis any diagnosis of pneumonia in an inpatient claim that was based on a secondary diagnosis or even in a principal diagnosis if there was evidence of an inpatient or long-term care stay in the previous 30 days. Productivity costs are estimated from the employer perspective and include only missed work time, not work time completed at a reduced productivity level. If illness affects labor force participation or has an effect on long-term disability, these productivity costs will not be captured. Finally, our method of estimating “excess costs” depends on the degree to which our matched control group is an accurate representation of the counterfactual of what the pneumonia patients would have experienced had they not contracted pneumonia. This assumption is not testable so our results must be interpreted in light of this uncertainty.

## Conclusion

In summary, we found that CAP is associated with substantial excess medical and productivity costs that can vary substantially by comorbidity group. Excess costs per case are highest for patients with a comorbidity of CHF and also elevated for patients with diabetes and COPD. Returns on prevention of pneumonia would therefore be higher in adults with these comorbidities than in adults without any of the selected comorbidities.

## Abbreviations

CAP: Community Acquired Pneumonia; HAP: Hospital Acquired Pneumonia; COPD: chronic obstructive pulmonary disease; CHF: Congestive Heart Failure; SE: Standard Error; SD: Standard Deviation; HPM: Thomson Reuters Health Productivity and Management database; CCI: Charlson Comorbidity Index.

## Competing interests

Dr. Polsky has received consulting fees from GlaxoSmithKline. Dr. Suaya is employed by the GlaxoSmithKline group of companies. At the time of this study, Suaya worked at GlaxoSmithKline Vaccines. Dr. Bonafede is a employee of Thompson Reuters.

## Authors’ contributions

All three authors have contributed to the conception, design, analysis, and interpretation of the data; the drafting and revising of the manuscript for important intellectual content; and have given final approval.

## Pre-publication history

The pre-publication history for this paper can be accessed here:

http://www.biomedcentral.com/1472-6963/12/379/prepub
